# A composite polymer nanoparticle overcomes multidrug resistance and ameliorates doxorubicin-associated cardiomyopathy

**DOI:** 10.18632/oncotarget.543

**Published:** 2012-07-10

**Authors:** Dipankar Pramanik, Nathaniel R. Campbell, Samarjit Das, Sonal Gupta, Venugopal Chenna, Savita Bisht, Polina Sysa-Shah, Djahida Bedja, Collins Karikari, Charles Steenbergen, Kathleen L. Gabrielson, Amarnath Maitra, Anirban Maitra

**Affiliations:** ^1^ The Sol Goldman Pancreatic Cancer Research Center, Johns Hopkins University School of Medicine, Baltimore, Maryland; ^2^ Department of Pathology, Johns Hopkins University School of Medicine, Baltimore, Maryland; ^3^ Department of Molecular and Comparative Pathobiology, Johns Hopkins University School of Medicine, Baltimore, Maryland; ^4^ Department of Oncology, Johns Hopkins University School of Medicine, Baltimore, Maryland; ^5^ Department of Internal Medicine 3, Center of Integrated Oncology Cologne-Bonn, University of Bonn, Germany; ^6^ Senior Scientist, Indian National Science Academy, New Delhi, India

**Keywords:** curcumin, doxorubicin, multidrug resistance

## Abstract

Acquired chemotherapy resistance is a major contributor to treatment failure in oncology. For example, the efficacy of the common anticancer agent doxorubicin (DOX) is limited by the emergence of multidrug resistance (MDR) phenotype in cancer cells. While dose escalation of DOX can circumvent such resistance to a degree, this is precluded by the appearance of cardiotoxicity, a particularly debilitating condition in children. *In vitro* studies have established the ability of the natural phytochemical curcumin to overcome MDR; however, its widespread clinical application is restricted by poor solubility and low bioavailability. Building upon our recently developed polymer nanoparticle of curcumin (NanoCurc or NC) that significantly enhances the systemic bioavailability of curcumin, we synthesized a doxorubicin-curcumin composite nanoparticle formulation called NanoDoxCurc (NDC) for overcoming DOX resistance. Compared to DOX alone, NDC inhibited the MDR phenotype and caused striking growth inhibition both *in vitro* and *in vivo* in several models of DOX-resistant cancers (multiple myeloma, acute leukemia, prostate and ovarian cancers, respectively). Notably, NDC-treated mice also demonstrated complete absence of cardiac toxicity, as assessed by echocardiography, or any bone marrow suppression, even at cumulative dosages where free DOX and pegylated liposomal DOX (Doxil®) resulted in demonstrable attenuation of cardiac function and hematological toxicities. This improvement in safety profile was achieved through a reduction of DOX-induced intracellular oxidative stress, as indicated by total glutathione levels and glutathione peroxidase activity in cardiac tissue. A composite DOX-curcumin nanoparticle that overcomes both MDR-based DOX chemoresistance and DOX-induced cardiotoxicity holds promise for providing lasting and safe anticancer therapy.

## INTRODUCTION

Resistance to chemotherapeutic drugs is a major impediment to a successful chemotherapeutic regimen. Cancer cells acquire drug resistance through a variety of mechanisms, not all of which are fully understood. Examples include host and tumor genetic alterations, epigenetic changes, changes in the tumor microenvironment, modification of the drug's cellular target, or blocking the drug's entry into the cell [1, 2]. Single drug resistant cells are often cross-resistant to other structurally and functionally different drugs, a phenomenon known as multidrug resistance (MDR) [3]. One key cause of acquired multidrug resistance is through energy-dependent efflux of cytotoxic agents through any of a 48-member family of ATP-binding cassette (ABC) transporters [2, 4, 5]. Such transmembrane efflux pumps, including MDR1 and MRP1, aid in tumor cell survival by actively removing chemotherapeutic agents from the cell's cytoplasm.

Resistance to chemotherapeutic drugs such as anthracyclines, vinca alkaloids, RNA-transporter inhibitors, and microtubule-stabilizing drugs can be associated with either single or multiple ABC transporters [6, 7]. For instance, resistance of metastatic tumors to the anthracycline doxorubicin (DOX) has been linked to overexpression of ABC transporters ABCB1 (MDR1/P-glycoprotein) [7], ABCC1 (MRP1) [8], ABCC2 (MRP2) [9, 10] and ABCG2 (MXR, BRCP) [11-13]. While dose escalation can circumvent treatment resistance to some degree, severe side effects including cardiotoxicity and bone marrow suppression limit the cumulative tolerable dose in patients. At a cumulative dose of 550 mg/m^2^ of DOX, 26% of patients develop congestive heart failure (CHF) [14], a condition that is lethal in approximately 50% of cases. The rate of CHF is further increased in pediatric patients, with the frequency of CHF in pediatric acute lymphoblastic leukemia (ALL) patients, for example, as high as 57% [15-17].

Towards the goal of overcoming multidrug resistance, several synthetic small molecules and antibodies targeted against MDR proteins have been tested *in vitro* and *in vivo* [18-22]; however, these inhibitors have largely failed in clinical trials due to toxicity and low serum stability [2]. Natural products are gaining attention in MDR inhibition due to their low cytotoxicity profiles. For example, the role of the phytochemical curcumin (derived from *Curcuma longa*) in inhibiting multiple MDR pumps in cancer cells has been widely studied [23-29], including in combination with DOX [30, 31]. Despite its promise, the full potential of treatments utilizing curcumin, either alone or in combination with chemotherapeutic drugs has not been realized in the clinic, primarily due to the poor systemic bioavailability of free curcumin outside the tubular lower GI tract [32].

We have recently developed a polymer nanoparticle formulation of curcumin (NanoCurc or NC) that significantly enhances the systemic bioavailability of this agent [33-35]. In order to harness the ability of curcumin in suppressing MDR and thus improve DOX efficacy in resistant cancer models, we synthesized a composite polymer nanoparticle of DOX and curcumin called ‘NanoDoxCurc’ (NDC) (Fig. S1). Our results confirm that curcumin encapsulated in a DOX-conjugated polymer nanoparticle can overcome DOX resistance in a variety of human and murine cancer cell lines *in vitro* as well as *in vivo*. Notably, we also find that systemic NDC shows no evidence of cardiotoxicity or bone marrow suppression, even at cumulative dosages at which such demonstrable adverse effects are readily observed in free DOX or Doxil®-treated mice, thus overcoming some of the greatest limitations of DOX-based chemotherapy.

## MATERIALS AND METHODS

All small-animal experiments described conformed to the guidelines of the Animal Care and Use Committee of the Johns Hopkins University. Mice were maintained in accordance with the guidelines of the American Association of Laboratory Animal Care. The doxorubicin resistant clones NCI/ADR and P388/ADR were obtained from the National Cancer Institute (Frederick, MD). The National Cancer Institute uses DNA fingerprinting for cell line authentication. PC-3A and parental PC-3 were the generous gift of Dr. William G. Nelson (Johns Hopkins University, Baltimore, MD), who generated the DOX-resistant clone [36]. RPMI8226/Dox and parental RPMI8226 were the generous gifts of Dr. William S. Dalton (Moffitt Cancer Center, Tampa, FL) who generated the DOX-resistant clone [37], and William Matsui (Johns Hopkins University, Baltimore, MD), respectively. DNA fingerprinting was used to authenticate cell lines not received directly from the NCI. All cells were cultured in RPMI 1640 medium supplemented with 10% FBS and pen/strep.

### Synthesis of NanoDoxCurc (NDC)

Doxorubicin was covalently grafted to the carboxylic acid residue of NVA622 polymer to make ‘NanoDox’ (ND). NVA622 polymer (200 mg) and EDCI (40 mg) were dissolved in distilled water (20 mL) and stirred for 30 min at room temperature. Doxorubicin (0.80 mg, 20 mg/mL in DMSO) was added to the reaction mixture and stirred for 6 h. The resulting reaction mixture was dialyzed for 12 h with exchange of fresh water every 2 h. The purified product (ND; DOX 1.4 μg/mg polymer *in vitro*, 2.5 μg/mg polymer *in vivo*) was lyophilized for use. Curcumin was encapsulated within the inner shell of ND or NVA622 as described previously [33] to make ‘NanoDoxCurc’ (NDC; curcumin 10 μg/mg polymer) or ‘NanoCurc’ (NC; curcumin 15 μg/mg polymer), respectively. The final concentration of drug was measured colorimetrically. For all *in vitro* studies, ND and NDC were reconstituted in cell culture medium to yield 25 μM DOX and 271 μM curcumin. NC was resuspended to yield 305 μM curcumin. For *in vivo* studies, drugs were reconstituted in sterile PBS.

### Trafficking of DOX into nucleus

Cells were seeded in 2-chambered slides one day prior to treatment. The next day either NDC or ND reconstituted in cell culture medium (500 μL/chamber, 10 mg/mL) were added to the appropriate chambers. After 2 h of treatment, medium was discarded, cells were fixed in 4% paraformaldehyde for 20 min, counterstained with DAPI, mounted, and examined using a confocal microscope (Zeiss) at 1000X final magnification.

### Rhodamine Exclusion Assay

Cells were seeded in a 6 well plate at 1.5×10^5^ cells per well and cultured overnight. The next day, media was changed to either 600 μL of cell culture medium or 600 μL of ND, NDC, or NC reconstituted as described above for 2 h. The cells were further incubated in fresh medium supplemented with 200 nM TMRM for 20 min. At the end of incubation, the cells were trypsinized, and suspended in PBS containing 2 mM EDTA and 2% FBS. The samples were analyzed in a BD FACSCalibur.

### Soft-Agar Assay

1×10^4^ cells were treated with ND, NDC, NC or medium alone for 2 h. Cells were washed and resuspended in 2 mL complete medium with 0.7% agar. This suspension was layered on solidified 2 mL base agar mixture of serum supplemented media and 1% agar on a 6-well plate. Subsequently, the plates were incubated at 37°C with 5% CO_2_ for 14 days to allow for colony growth. The plates were then stained and colonies counted on ChemiDoc XRS instrument (Bio-Rad, Hercules, CA). Results are presented relative to the number of viable cells by cell survival assay.

### Xenograft Studies

Flanks of 5-6 week old male athymic *nu/nu* mice (Harlan Laboratories, Indianapolis, IN) were injected with 5×10^6^ PC-3A or RPMI8226/Dox cells suspended in a total volume of 200 μL [PBS/Matrigel (BD Biosciences), 1:1 (v/v), pre-chilled to 4°C]. After one week, twenty mice per tumor type with successfully engrafted xenografts were randomized into four cohorts of five animals each and administered i.p. (*i*) vehicle, (*ii*) ND (6 mg/kg DOX equivalent), (*iii*) NC (30 mg/kg curcumin equivalent), or (*iv*) NDC (6 mg/kg DOX equivalent; 24 mg/kg curcumin equivalent) twice every three days. Tumor size (*ab*^2^/2; *a* and *b* are the long and short axes of the tumor) and body weight were measured weekly. At the culmination of treatment, visceral organs and tumor tissues were harvested and either preserved in 10% neutral buffered formalin or snap frozen.

### P388/Dox Ascites

P388/Dox DOX-resistant ascites were implanted intraperitoneally in two B6D2F1 (BDF1) mice (6 weeks, Harlan Laboratories, Indianapolis, IN). After 7 days, ascitic fluid was collected via syringe and injected into 24 BDF1 mice. The following day, mice were randomized into three arms receiving daily either *(i)* ND at a dose of 6 mg/kg DOX equivalent, *(ii)* NDC at a dose of 6 mg/kg DOX equivalent and 24 mg/kg curcumin equivalent, and *(iii)* vehicle. After 6 days (following the first death in the vehicle arm) treatment was terminated and mice followed for survival for the remainder of the study.

### Cumulative Dose Toxicity

4-5 week old C57BL/6J mice (5 mice per arm; Harlan Laboratories, Indianapolis, IN) were injected intravenously with free DOX, Doxil, ND, NDC or PBS at 9mg/kg doxorubicin equivalent once weekly for 4 weeks. One week following the last injection echocardiography was performed and blood was collected by cardiac puncture. Heart tissue was harvested and snap frozen.

### Quantification of Total Glutathione

Total glutathione was measured using an NADPH linked enzymatic colorimetric assay by measuring the absorbance at 412 nm. Reagents were obtained from Sigma (CS0260; St. Louis, MO). Briefly, 10 mg of snap-frozen heart was dissolved in 1 mL of 5% 5-Sulfosalicylic Acid (SSA) on ice for 10 minutes, and then centrifuged at 10,000 × g for 10 minutes. Supernatants were collected and analyzed according to manufacturer's protocol. Heart lysates from age-matched untreated C57BL/6J mice were used as controls.

### Glutathione peroxidase (GPx) Activity Assay

GPx activity was measured using an NADPH linked enzymatic assay by measuring the decrease in NADPH absorbance at 340 nm. Reagents were obtained from Sigma (CGP1; St. Louis, MO), with mitochondrial fractions containing GPx isolated from hearts from different treatment groups.

### Statistical Analysis

Two-tailed t test and survival analysis were performed using Prism version 5.01 (GraphPad Software, Inc.). *p*<0.05 was regarded as statistically significant. Diagrams show means and SDs.

## RESULTS

### NDC formulation overcomes MDR pump induced DOX efflux in vitro

A composite formulation of DOX and curcumin was synthesized by covalently conjugating DOX to the carboxylic acid moiety on the surface of the amphiphilic polymer (NanoDox; ND), followed by encapsulating curcumin within its hydrophobic core (NanoDoxCurc; NDC) (Fig. S1). To test the ability of NDC to overcome MDR, thus allowing DOX to accumulate in the cell and be trafficked to the nucleus, we chose three independent DOX resistant human cancer cell lines (NCI/ADR [ovarian], PC-3A [prostate] and RPMI8226/Dox [multiple myeloma]) expressing high levels of distinct MDR proteins - MDR1 and MRP1 (Fig. S2a). Two of the parental cell lines (PC-3, RPMI8226) were available as controls.

**Figure 1:**
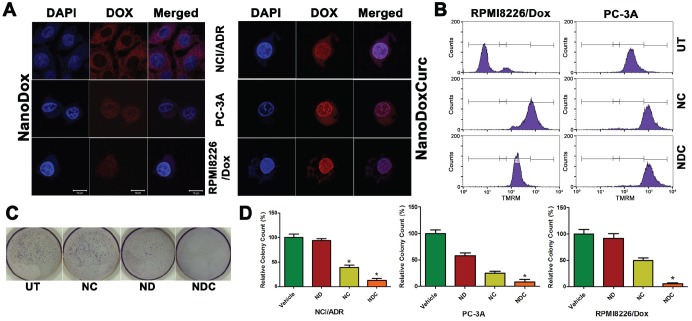
Trafficking of DOX into the nucleus and cytotoxicity are enhanced by curcumin in DOX resistant clones (a) Nuclear accumulation of ND and NDC as measured by doxorubicin fluorescence. Enhanced nuclear accumulation could be observed following treatment with NDC (right panel) compared with ND (left panel). Scale bar is 10μm. (b) Rhodamine accumulation as measured by flow cytometry. Accumulation of more rhodamine dye in NDC and NC treated lines indicated inhibition of MDR protein function. (c,d) NDC significantly inhibits clonogenicity as measured by soft agar colony formation. Colony counts and representative NCI/ADR plates are shown (N=3, *p<0.05).

We initially assessed whether the curcumin-containing NDC formulation allowed accumulation of DOX inside the nucleus as measured by doxorubicin fluorescence. In parental, non-DOX resistant cell lines ND co-localized with DAPI as expected, indicating accumulation of ND inside the nucleus (Fig. S2b). When resistant NCI/ADR, PC-3A, and RPMI8226/Dox cell lines were treated with ND alone, very little nuclear DOX fluorescence signal was observed, indicating poor nuclear accumulation of DOX (Fig. 1a, Left Panel). In stark contrast, treatment with NDC dramatically induced nuclear accumulation in DOX resistant cell lines, indicating the ability of co-treatment with curcumin to promote nuclear uptake of DOX (Fig. 1a, Right Panel).

To further confirm the ability of curcumin to reduce drug resistance by inhibiting drug effusion, we evaluated the exclusion of rhodamine dye by flow cytometry, a standard assay to assess MDR function [38], in MDR1- and MRP1-expressing RPMI8226/Dox and MRP1-expressing PC-3A cell lines. As seen in untreated controls, rhodamine dye is very efficiently removed from the cytoplasm (Fig. 1b). In both cell lines, treatment with either NC or NDC resulted in enhanced rhodamine accumulation, confirming the potential of curcumin to overcome ABC transporter function in MDR cell lines.

### NDC significantly reduces viability and clonogenic growth of DOX-resistant human cancer cells

To test whether the NDC formulation increases the cytotoxic effects of DOX in DOX-resistant clones, we evaluated cell viability following treatment with ND, NC and NDC for 48 hours. All three lines were nearly completely refractory to ND alone, and only minimal sensitivity to NC was observed in PC-3A and RPMI8226/Dox. In contrast, NDC treatment resulted in significant decreases in proliferation in all three DOX-resistant cell lines (Fig. S2c). In a similar fashion, treatment with NDC significantly reduced clonogenicity, with ND alone showing only mild to moderate decreases in colony count in PC-3A (Fig. 1c,d). Interestingly, NC alone showed greater potency than ND in all three DOX-resistant cell lines.

### NDC significantly inhibits the growth of DOX-resistant human tumor xenografts and improves survival of mice bearing syngeneic leukemic ascites

**Figure 2:**
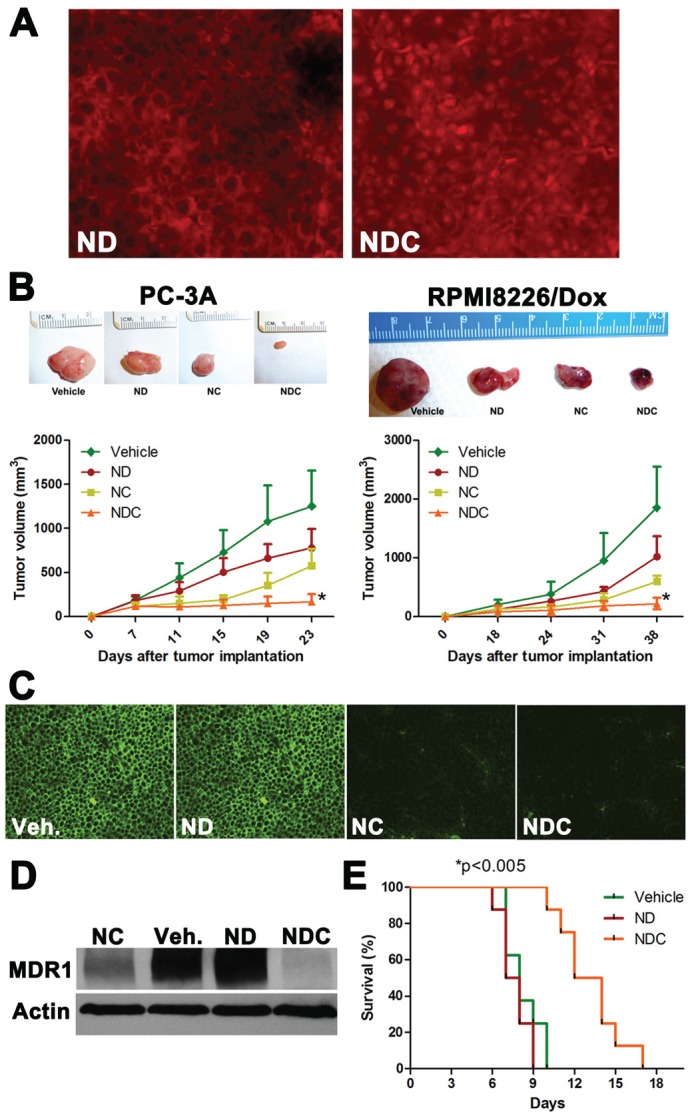
NDC overcomes DOX resistance *in vivo* (a) Representative tumor sections from RPMI8226/Dox xenografts were examined by fluorescence microscopy for nuclear accumulation of DOX by doxorubicin fluorescence. (b) NDC significantly inhibits the growth of subcutaneous DOX-resistant cancer xenografts PC-3A and RPMI8226/Dox. Representative excised xenograft tumors from each of the four arms are illustrated. NDC significantly blocked tumor growth compared to either ND or NC (N=5, *p<0.005). Post treatment levels of MDR1 expression were measured by (c) immunofluorescence and (d) western blot in RPMI8226/Dox xenografts. (e) BDF1 mice bearing P388/ADR DOX-resistant ascites were treated with vehicle, ND, or NDC. A greater than 50% increase in survival was observed in NDC treated mice compared to ND or vehicle treated mice (N=8, *p<0.005).

PC-3A and RPMI8226/Dox DOX-resistant clones were implanted subcutaneously in the right flank of athymic nude mice, and treated with vehicle, ND, NC, or NDC. *In vivo* nuclear accumulation of DOX was measured by fluorescence microscopy in formalin-fixed paraffin-embedded RPMI8226/Dox xenograft sections (Fig. 2a). The presence of DOX was observed in the cytoplasmic compartment in ND-treated xenografts; however, marked nuclear accumulation of DOX was only observed in sections from NDC-treated tumors. In both xenograft models, treatment with either ND or NC alone significantly reduced the rate of growth of tumor by approximately 50% (*p*<0.05 and *p*<0.01, respectively). Demonstrating the benefit of the composite formulation, treatment with NDC yielded a greater than 90% reduction in tumor growth (Fig. 2b) (*p*<0.005; vs. all other treatment groups). Importantly, the body weight of animals treated with ND or NDC for 2-3 weeks was not significantly different as compared to controls, suggesting a favorable toxicity profile at therapeutically relevant doses (Fig. S3a). Histological analysis of sections from treated tumors in both models showed significant necrotic regions in NDC-treated tumors, and to a lesser extent in NC-treated cases (Fig. S3b,c). Additionally, staining for the cell proliferation marker Ki67 showed markedly lower proliferation in RPMI8226/Dox xenografts treated with NDC as compared to ND, NC, or untreated control (Fig. S3d). Immunofluorescence and western blot analysis of RPMI8226/Dox xenografts indicated greatly reduced expression of MDR1 in NC- and NDC-treated xenografts (Fig. 2c,d).

In a syngeneic model of DOX resistance, we evaluated whether NDC increases the survival of wild-type BDF1 mice injected intraperitoneally with murine P388/ADR DOX-resistant ascites (Fig. 2e). The P388/ADR is a highly aggressive DOX resistant clone derived from a murine acute leukemia [39]. Treatment with ND showed no survival benefit over vehicle controls, with both groups showing a median survival of approximately 8 days. In contrast, a significant increase in median survival of greater than 50% was observed upon treatment with NDC, with mice surviving a median of 13 days (*p*<0.005).

### Systemic NDC and ND exhibit minimal cardiotoxicity and bone marrow suppression as compared to Doxorubicin and Doxil®

**Figure 3:**
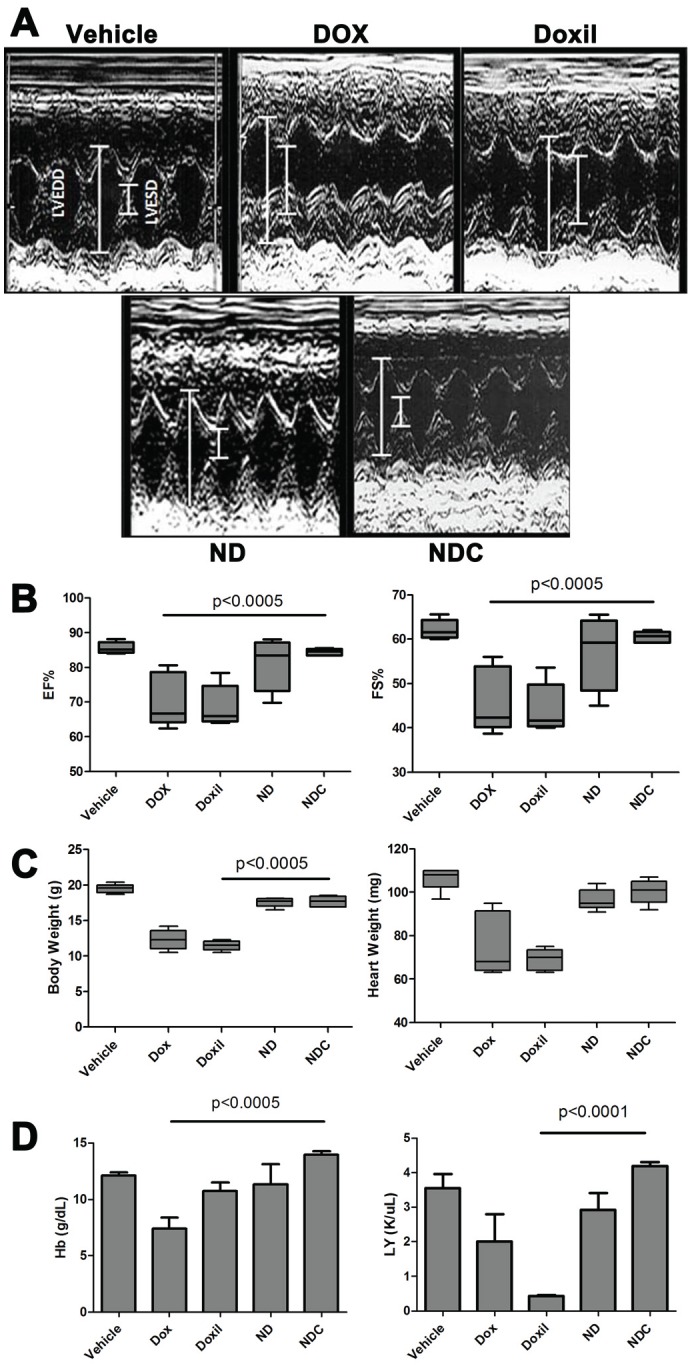
Doxorubicin formulation NDC has no observable cardiotoxicity or bone marrow suppression Following 4 weeks of receiving weekly doses of either vehicle, free DOX, Doxil, ND, or NDC, various cardiac parameters were examined by echocardiography and molecular analysis. (a) Representative echocardiogram graph and (b) cardiac parameters plotted graphically (N=5). (c) Body weight and heart weight, and (d) hemoglobin and leukocyte counts from each treatment arm (N=5).

A major dose limiting factor for DOX-based regimens in the clinic is the development of cardiotoxicity, especially in the pediatric population. We compared the toxicities of both the ND and NDC formulations with those of free DOX and Doxil®, a commercially available pegylated liposomal formulation of DOX. C57BL/6 wild-type mice were injected intravenously with buffered saline, free DOX, Doxil, ND, or NDC once every week for 4 weeks at comparable cumulative dosages (9 mg/kg DOX equivalent). One week after the last dose, cardiac function of the mice was measured by echocardiography (Fig. 3a). DOX and Doxil®-treated mice showed a significant increase in all of the assessed parameters considered detrimental to cardiac function, including left ventricular end systolic dimension (LVESD), interventricular septal wall thickness at end diastole (IVSD), left ventricular posterior wall thickness at end diastole (LVPWTD); the results of which were an objective decrease of fractional shortening (FS), and ejection fraction (EF) in both cohorts. In contrast, mice treated with ND, and in particular those receiving NDC, demonstrated minimal evidence of the aforementioned signs of cardiotoxicty, as compared to free DOX or Doxil® (Fig. 3b). In fact, the echocardiographic parameters were essentially overlapping in NDC and vehicle-treated mice.

Both DOX and Doxil® treatment substantially reduced mouse body weight and heart weight by more than 40% (Fig. 3c); however, ND- and NDC-treated mice showed no significant change in body and heart weight relative to controls. Blood samples were collected from experimental animals by cardiac puncture, and hearts were collected for histological analysis and molecular studies. Hemoglobin (Hb) levels dropped from an average of 12.5 g/dL in control mice to an average of 7.5 g/dL in DOX-treated mice, and lymphocyte counts were significantly reduced in Doxil®-treated animals, indicating both anemia and severe lymphocytopenia (Fig. 3d). By contrast, there were no significant alterations in hematological parameters in ND- and NDC-treated mice compared to vehicle controls, indicating minimal bone marrow suppression.

**Figure 4:**
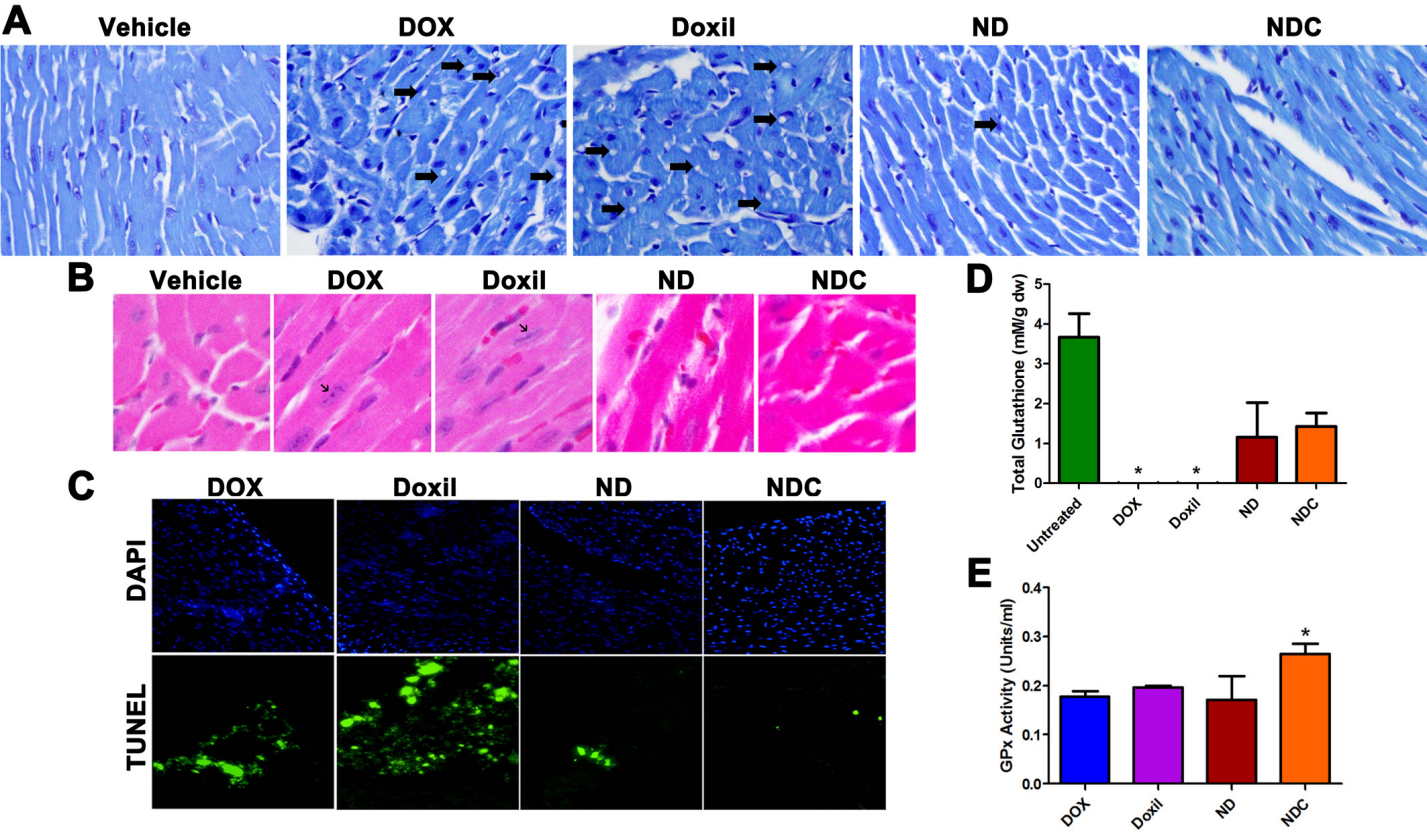
Doxorubicin formulation NDC has no observable cardiotoxicity (a) Toluidine blue stained heart sections showed the presence of cardiomyopathy (holes, indicated by arrows) in DOX and Doxil treated groups, which was notably absent in ND and NDC treated mice. (b) H&E stained heart sections indicated the presence of hypertrophic cardiac cells in DOX and Doxil treated mice (black arrows), which was also absent in ND and NDC treated mice, as well as controls. (c) TUNEL assay of heart sections indicating increased presence of apoptotic cardiac cells in DOX and Doxil treated groups. (d, e) Enzymatic assay analysis of total glutathione levels and glutathione peroxidase (GPx) activity in heart lysates.

Histological assays and TUNEL staining were also performed on heart cryosections to examine for indications of doxorubicin-induced apoptosis and cardiomyopathy. Toluidine blue-stained heart sections from DOX and Doxil®-treated animals demonstrated widespread lesions consistent with acute cardiomyopathy in these groups (Fig. 4a, arrows). In contrast, sections from ND- and NDC-treated mice were indistinguishable from those of vehicle-treated controls. Additionally, analysis of H&E stained sections revealed the presence of hypertrophic cardiac cells—characterized by elongated nuclei—in both DOX- and Doxil®-treated mice; however, no such lesions were found in sections from ND and NDC groups (Fig. 4b). Finally, TUNEL staining indicated widespread apoptosis in cardiac cells in both DOX- and Doxil®-treated mice. In contrast, few apoptotic cells were observed in ND treated mice, and no apoptotic cells were observed in NDC and vehicle treated groups (Fig. 4c).

The underlying basis for cardiomyocyte damage with DOX exposure is considered to be aberrant intracellular oxidative stress, due to DOX-induced reactive oxygen species (ROS) [40]. Our recent studies with nanoparticulate curcumin have confirmed its ability to ameliorate oxidative damage to non-neoplastic tissues, such as in hepatocytes and neuronal cells [34, 35], through induction of a favorable intracellular redox environment. To evaluate the degree of oxidative stress in treated mice, total glutathione levels, and activity of the antioxidant glutathione peroxidase (GPx) were quantified using lysates prepared from cardiac tissues. In both DOX- and Doxil®-treated groups, cardiac glutathione levels were nearly undetectable (Fig. 4d; *p*<0.05). In contrast, glutathione levels in both ND- and NDC-treated groups were not significantly different than in untreated mice (*p*=0.5). Further, only the NDC-treated mice showed significantly higher levels of GPx activity than all other treatment groups (Fig. 4e), underscoring the enhanced anti-oxidant capacity resulting from the inclusion of curcumin concurrently with DOX in the composite formulation.

## DISCUSSION

Multiple drug resistance caused by overexpression of ATP-binding cassette (ABC) transporters is a major impediment in cancer chemotherapy [2]. Current approaches to overcome MDR include a focus on drug discovery, with, in many cases, an end goal of combination therapy [2]. Although multiple lines of evidence have established curcumin as an inhibitor of ABC-transporter function [31, 41], its use *in vivo* has been limited by the poor systemic bioavailability of this hydrophobic small molecule. Following our recent development of a highly-bioavailable nanoparticle-encapsulated formulation of curcumin (NanoCurc) [33], we sought to develop a composite curcumin-doxorubicin nanoparticle, NanoDoxCurc (NDC), which could overcome MDR protein function and provide more efficacious therapy for patients in an important step forward in improving overall cancer survival. As an additional benefit, curcumin, which is known to induce a favorable intracellular redox environment [42], might be expected to reduce cardiac toxicity in such a composite nanoparticle, opening the possibility of increased safety at higher cumulative doses of DOX.

Following the synthesis of NDC by covalent modification of the existing NanoCurc (NC) formulation, we began investigating the *in vitro* effects of this composite nanoparticle. We observed that curcumin strongly repressed the MDR phenotype in DOX-resistant cancer cell lines that constitutively overexpress the MDR proteins MDR1 and MRP1, allowing robust nuclear uptake of DOX. This was in contrast to the previously described DOX nuclear exclusion pattern characteristic of MDR cells, which was observed in ND-treated cells [43]. The inhibition of the MDR phenotype by NDC was accompanied by significant decreases in both *in vitro* cell viability and colony formation on soft agar. Of note, we observed the improved efficacy of NDC over ND in cancer lines with distinct patterns of MDR protein overexpression (MDR1 or MRP1, respectively), underscoring the potentially broad utility of this strategy across cancer types.

Based on the *in vitro* data, we next assessed the efficacy of the NDC formulation *in vivo* using DOX-resistant human cancer xenografts. Compared to vehicle, NC, or ND alone, NDC significantly inhibited subcutaneous tumor growth in PC-3A and RPMI8226/Dox xenografts, yet the treated mice showed no evidence of toxicity, maintaining body weight and demonstrating no overt behavioral changes throughout the duration of treatment. Interestingly, while both ND and NC each showed a degree of tumor growth inhibition, the composite nanoparticle NDC showed nearly complete growth inhibition (>90%) over the duration of the study. Comparable nuclear DOX uptake patterns to those observed *in vitro* were found in NDC-treated xenografts, indicating the suppression of MDR phenotype *in vivo* by curcumin, which was also confirmed by *ex vivo* immunofluorescence and Western blot analysis of treated tumors. To further evaluate the therapeutic efficacy of NDC, we utilized BDF1 wild-type mice injected with MDR-overexpressing P388 DOX-resistant ascites [44], which is a more biologically relevant preclinical model than subcutaneous xenografts. As expected, this model was completely refractory to ND treatment, with no observed change in survival. In contrast, treatment with NDC markedly increased the median survival by more than 50% as compared to ND or vehicle treatment. Taken together these results demonstrate the ability of nanoparticle-delivered curcumin to effectively overcome MDR *in vivo* by inhibiting ABC-transporter expression, restoring the otherwise excellent therapeutic efficacy of DOX in a variety of clinically relevant model systems.

In the treatment of malignancies with DOX, the occurrence of cardiotoxicity is dose-dependent, limiting the cumulative dose a patient may receive, and thus limiting the therapeutic efficacy of the drug [45]. Because the mechanism of DOX-induced cardiotoxicity is independent of its mechanism of action in tumors [40], there exists the potential to selectively block the systemic toxicity of DOX without affecting its therapeutic benefit. We postulated that a composite nanoparticle formulation of DOX and curcumin (NDC) would not only circumvent the MDR phenotype in tumor cells, but also attenuate oxidative stress induced damage in extra-tumoral tissues, such as the heart. To evaluate this hypothesis, we investigated the cardiotoxicity of NDC in a model of high cumulative dose toxicity in C57BL/6 wild-type mice as evaluated by echocardiography. Mice treated with either DOX or Doxil® showed unequivocal signs of decrease in cardiac function. In particular, significant decreases in both ejection fraction (EF) and fractional shortening (FS) were observed, key clinical indicators of impaired myocardial function [15, 45]. In contrast, mice treated with comparable cumulative dosages of ND or NDC showed minimal impairment of cardiac function, as assessed by echocardiographic parameters. Similarly, hemoglobin and leukocyte counts were found to be reduced by DOX and Doxil®; in contrast, both ND and NDC treated mice showed counts similar to controls, indicating absence of bone marrow suppression as well. One possible explanation for the protection afforded is that curcumin causes cell cycle arrest in bone marrow cells, sparing them from the cyctotoxic effects of DOX in a manner analogous to cyclotherapy [46, 47]. Further studies are required to fully elucidate the mechanism by which NDC spares mice from bone marrow suppression; however, such an approach would be of high clinical utility.

As the major mechanism of doxorubicin-induced cardiotoxicity is oxidative stress [40, 45], we evaluated glutathione levels and glutathione peroxidase (GPx) activity in cardiac tissue. Not surprisingly, reduced glutathione levels were observed in cardiac tissue of DOX- and Doxil®-treated mice, indicating that both treatments induce oxidative stress within cardiomyocytes and depleted intracellular anti-oxidant reserves. In contrast, ND- and NDC-treated mice maintained glutathione levels comparable to that observed in untreated mice, while an additional indicator of enhanced anti-oxidant function—namely, increased GPx activity—was observed solely in the NDC-treated mice. Thus, nanoencapsulation of DOX (i.e., ND) is sufficient to provide a reasonable degree of cardioprotection compared to comparable dosages of free DOX or Doxil®, but it is only the composite formulation (NDC) that induces both a favorable redox environment in non-neoplastic tissues, while concomitantly overcoming therapeutic resistance in the neoplastic cells.

In conclusion, we have designed a composite polymeric nanoparticle, which has doxorubicin covalently bound to the surface of the nanoparticle, and curcumin encapsulated within its hydrophobic core. Due to the presence of curcumin, a potent inhibitor of MDR, this composite nanoparticle (NDC) can unequivocally overcome multidrug resistance as demonstrated in multiple *in vivo* models of DOX-resistant human and murine cancers. Additionally, NDC shows significantly reduced cardiotoxicity in mice receiving high cumulative doses of DOX, due to the attenuation of oxidative stress in systemic tissues by curcumin. Such composite nanoparticles have great promise for clinical translation, as they directly address multiple challenges by both overcoming resistance and enhancing safety, effectively “killing two birds with one stone”.

## Supplementary Figures and Methods




