# Correction: A composite polymer nanoparticle overcomes multidrug resistance and ameliorates doxorubicin-associated cardiomyopathy

**DOI:** 10.18632/oncotarget.28853

**Published:** 2026-04-24

**Authors:** Dipankar Pramanik, Nathaniel R. Campbell, Samarjit Das, Sonal Gupta, Venugopal Chenna, Savita Bisht, Polina Sysa-Shah, Djahida Bedja, Collins Karikari, Charles Steenbergen, Kathleen L. Gabrielson, Amarnath Maitra, Anirban Maitra

**Affiliations:** ^1^The Sol Goldman Pancreatic Cancer Research Center, Johns Hopkins University School of Medicine, Baltimore, Maryland; ^2^Department of Pathology, Johns Hopkins University School of Medicine, Baltimore, Maryland; ^3^Department of Molecular and Comparative Pathobiology, Johns Hopkins University School of Medicine, Baltimore, Maryland; ^4^Department of Oncology, Johns Hopkins University School of Medicine, Baltimore, Maryland; ^5^Department of Internal Medicine 3, Center of Integrated Oncology Cologne-Bonn, University of Bonn, Germany; ^6^Senior Scientist, Indian National Science Academy, New Delhi, India. π Deceased; ^*^Constitutes equal contribution

**This article has been corrected:** This article has been corrected: It was noted that the immunofluorescence image for ‘NanoDox’ (ND) in Figure 2C was incorrectly selected, resulting in identical images being shown for both the vehicle (Veh) and ND post-treatment levels of MDR1 expression in RPMI8226/Dox xenografts.

The authors have provided a corrected Figure 2C using the original image for the ND post-treatment level of MDR1 expression. The replacement of this image does not alter the study’s conclusions. The authors sincerely apologize for any inconvenience this may cause to readers.

Original article: Oncotarget. 2012; 3:640–650. 640-650. https://doi.org/10.18632/oncotarget.543

**Figure 2:**
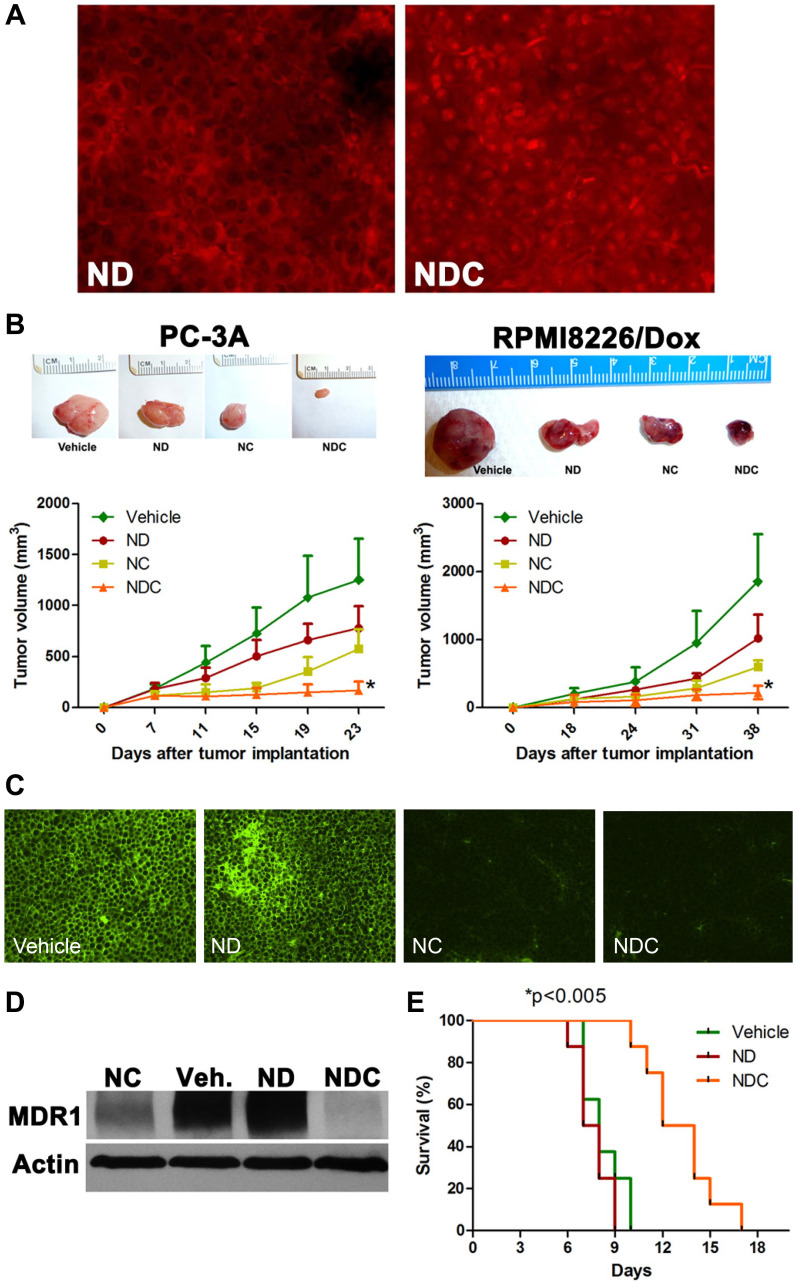
NDC overcomes DOX resistance *in vivo*. (**A**) Representative tumor sections from RPMI8226/Dox xenografts were examined by fluorescence microscopy for nuclear accumulation of DOX by doxorubicin fluorescence. (**B**) NDC significantly inhibits the growth of subcutaneous DOX-resistant cancer xenografts PC-3A and RPMI8226/Dox. Representative excised xenograft tumors from each of the four arms are illustrated. NDC significantly blocked tumor growth compared to either ND or NC (*N* = 5, ^*^p < 0.005). Post treatment levels of MDR1 expression were measured by (**C**) immunofluorescence and (**D**) western blot in RPMI8226/Dox xenografts. (**E**) BDF1 mice bearing P388/ADR DOX-resistant ascites were treated with vehicle, ND, or NDC. A greater than 50% increase in survival was observed in NDC treated mice compared to ND or vehicle treated mice (*N* = 8, ^*^p < 0.005).

