# Supplementary material for: A composite polymer nanoparticle overcomes multidrug resistance and ameliorates doxorubicin-associated cardiomyopathy
Source: Oncotarget. 2012 Jul 10;3(6):640–50. doi: 10.18632/oncotarget.543 (PMC3442295; doi:10.18632/oncotarget.543)
Supplement: Supplementary file 1 [file oncotarget-03-640-s001.pdf]

A composite polymer nanoparticle overcomes multidrug resistance and ameliorates doxorubicin-associated cardiomyopathy - Pramanik et al

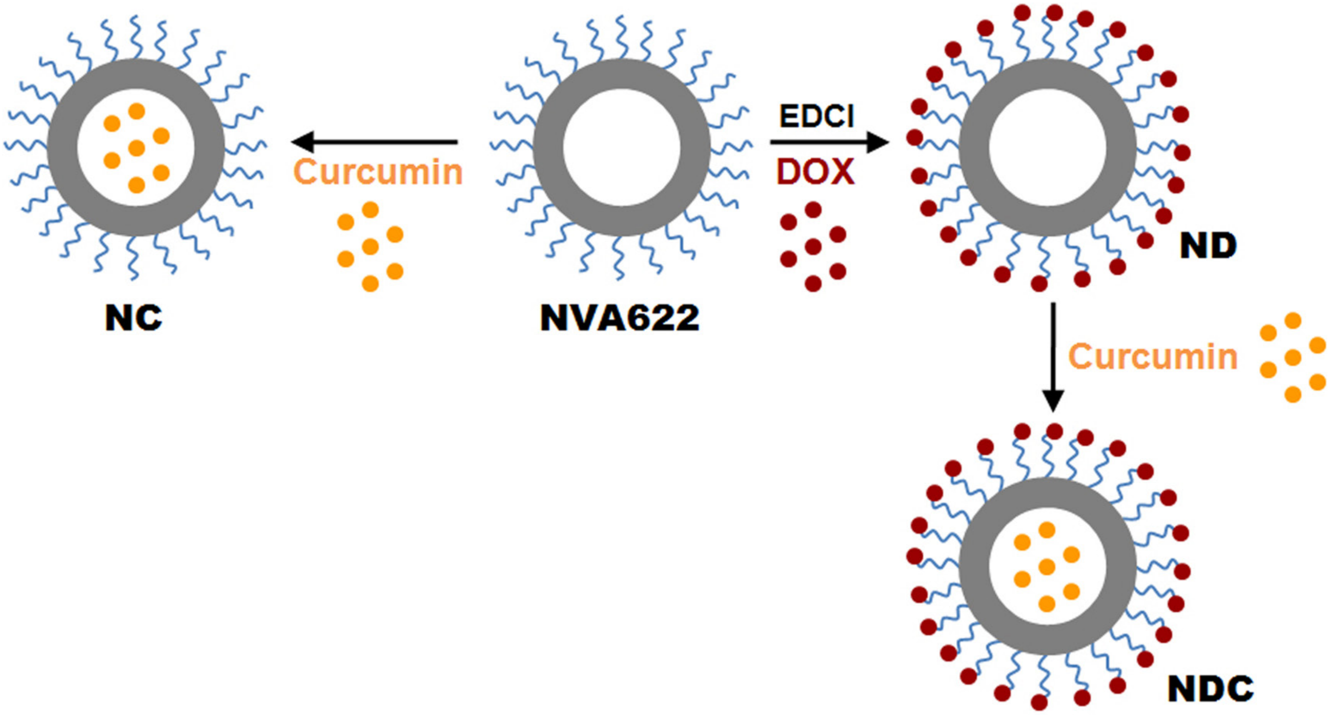

Figure S1: Schematic of NanoCurc (NC), NanoDox (ND), and NanoDoxCurc (NDC) synthesis from NVA622.

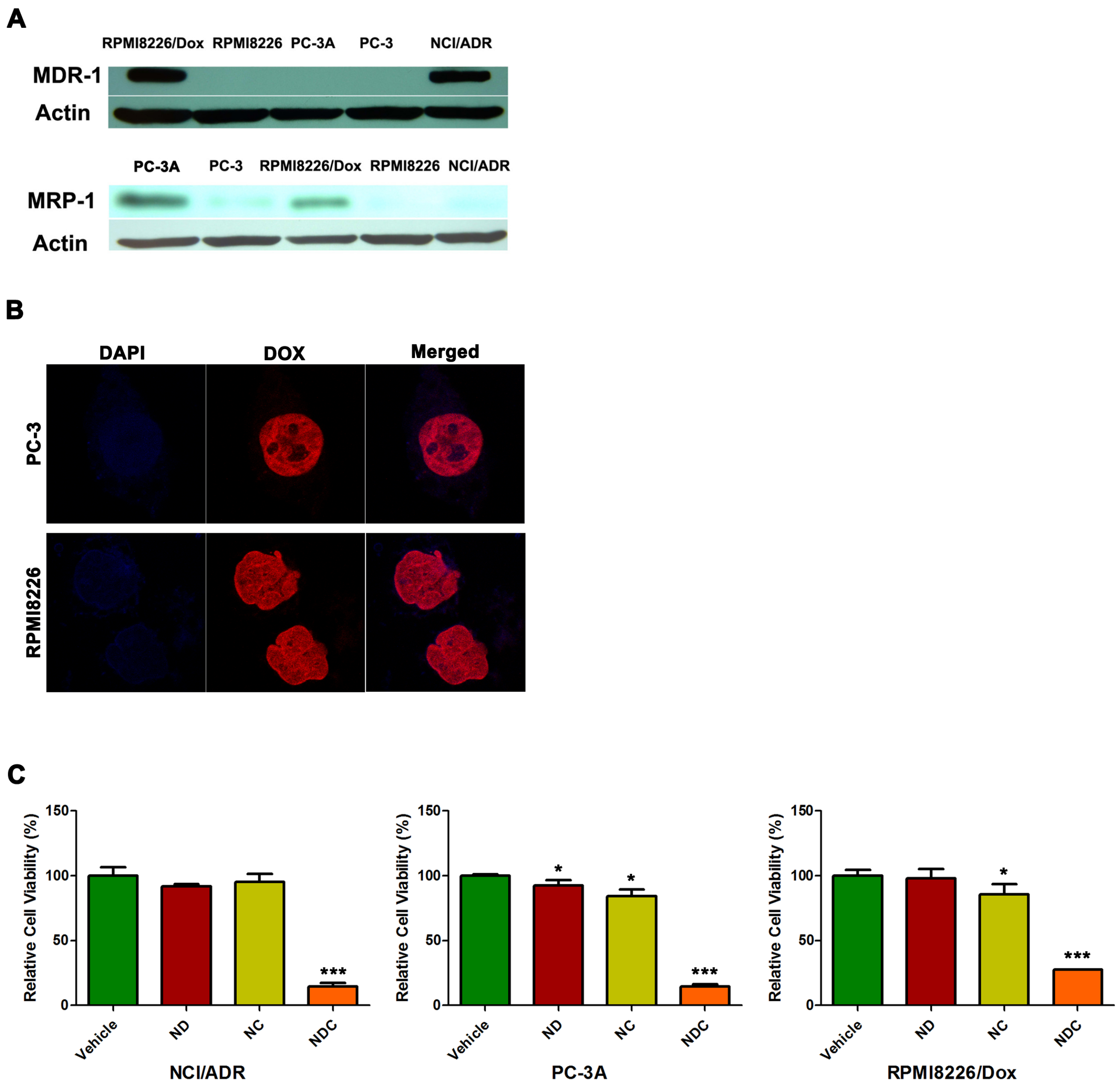

Figure S2: *In vitro* analysis of ND and NDC formulations. (a) Western blot analysis of MDR1 and MRP1 in DOX resistant and parental cell lines shows increased MDR1 and MRP1 expression in resistant clones. (b) Nuclear accumulation of ND in parental PC-3 and RPMI8226 cell lines as measured by doxorubicin fluorescence. (c) Cytotoxic efficacy of NanoDox (ND), NanoCurc (NC), and NanoDoxCurc (NDC) towards DOX resistant clones. NDC significantly inhibited the growth of all three DOX resistant cancer cell lines relative to control, ND, and NC (N=3; ***p<0.0001; *p<0.05 vs. vehicle).

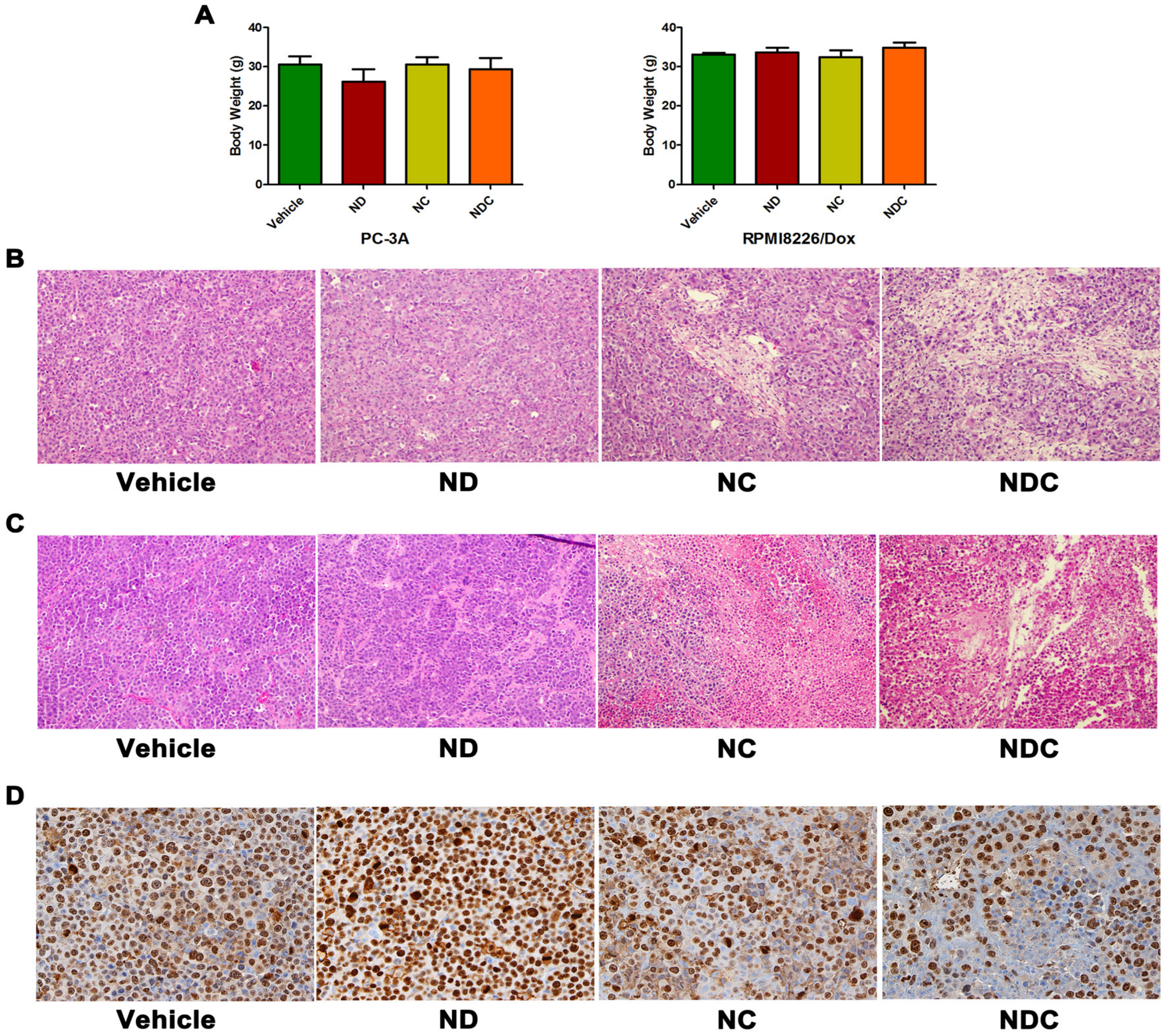

Figure S3: Analysis of DOX-resistant xenografts treated with ND, NC, and NDC. (a) No reduction in body weight was observed over the course of treatment (N=5). In PC-3A (b) and RPMI8226/Dox (c) xenografts, significant necrotic regions were observed in NDC treated H&E stained tumor sections. (d) In RPMI8226/Dox xenografts, significant downregulation of proliferation marker Ki-67 was observed in NDC treated tumor sections by immunohistochemical staining.

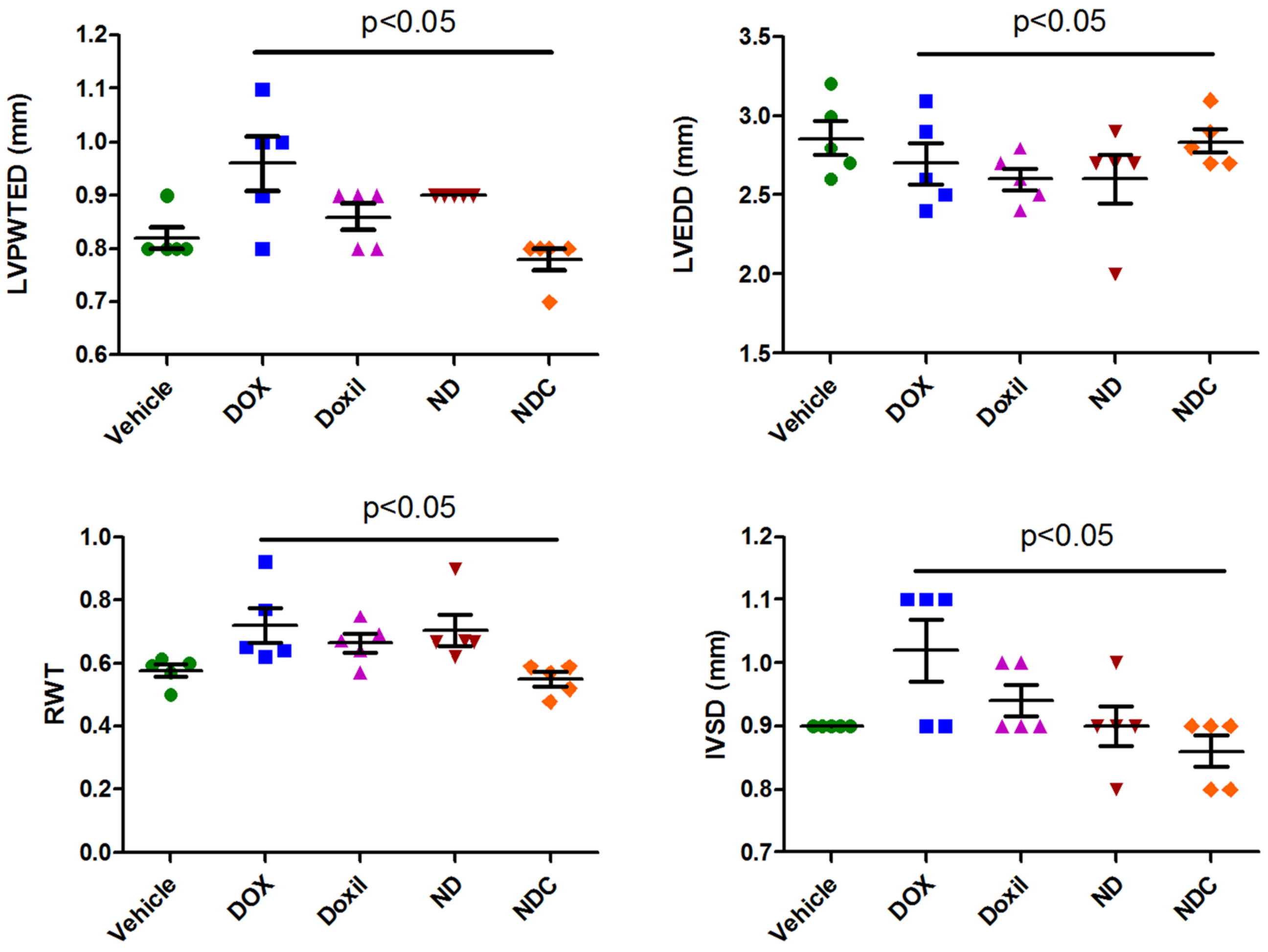

Figure S4: Parameters from echocardiograph plotted graphically (N=5).
