# Supplementary material for: A composite polymer nanoparticle overcomes multidrug resistance and ameliorates doxorubicin-associated cardiomyopathy
Source: Oncotarget. 2012 Jul 10;3(6):640–50. doi: 10.18632/oncotarget.543 (PMC3442295; doi:10.18632/oncotarget.543)
Supplement: Supplementary file 2 [file oncotarget-03-640-s002.pdf]

Supplementary Information

Materials and Methods

NVA622 polymer was purchased from Lakeshore Biomaterials. Doxorubicin and EDCI were purchased from Sigma-Aldrich. Curcumin was purchased from Sabinsa. Anti-MDR1, and MRP1 antibodies were procured from Santa Cruz Biotech. Anti-p65 and anti-MIB1 were purchased from Cell Signaling and Ventana Biological Systems, respectively.

Cell Survival Assay

A panel of three DOX-resistant cancer cell lines NCI/ADR (breast cancer), PC-3A (prostate cancer), and RPMI8226/Dox (myeloma) were cultured in 96-well plates and treated with ND (10 mg/mL), NDC (10 mg/mL), and NC (7.5 mg/mL) for 2 h. Following treatment the plates were washed with PBS and the cells were cultured in fresh growth medium for a further 48 h. Growth inhibition was measured by CellTiter 96® Aqueous Cell Proliferation Assay (Promega) according to manufacturer's protocol.

Western Blot

Cell pellets were lysed with RIPA buffer, and 50 µg of protein per sample was separated on a 4-20% SDS-polyacrylamide gradient gel (SDS-PAGE, Invitrogen). Proteins were transferred onto a nitrocellulose membrane (Hybond-ECL, Amersham Biosciences, NJ) and blocked for 1 h with 5% non-fat milk in PBS containing 0.5% Tween-20 (PBS-T). Blots were then incubated with appropriate primary and HRP-conjugated secondary antibodies at 1:500 and 1:5000 dilutions, respectively. Chemiluminescent signal was developed with ECL substrate (Thermo Scientific). Anti-actin antibody (dilution of 1:2000) was used as an internal control for protein loading.

Mouse Echocardiography

4-5 week old C57BL/6J mice (5 mice per arm; Harlan Laboratories, Indianapolis, IN) were injected intravenously with free doxorubicin, Doxil, ND, NDC, or PBS buffer at 9mg/kg doxorubicin equivalent once weekly for 4 weeks. One week following the last injection echo cardiogram was performed. All measurements were performed using the leading-edge method, as recommended by the American Society of Echocardiography [1]. To perform echocardiography on conscious animals [2], mice were gently held in supine position in the palm of the hand. The left hemithorax was shaved and a 1-2 mm thick layer of prewarmed hypoallergenic ultrasonic transmission gel (Parker Laboratories, Fairfield, New Jersey) was applied to the thorax. Transthoracic echocardiography was performed using a Hewlett-Packard Sono 5500 ultrasound machine with a 15 MHz transducer. Images were stored on a 1.2 GB magnetic optical disk (Hewlett Packard) and T120 VHS tape. Two-dimensional and left ventricle M-mode measurements were taken in two separate 3-4 min sessions. The heart was first imaged in two-dimensional mode in the parasternal short axis view at a sweep speed of 150 mm/s. From this mode, an M-mode cursor was positioned perpendicular to the inter-ventricular septum and the left ventricular posterior wall thickness (LVPW) at the level of the papillary muscles. From the M-mode, the left ventricular wall thickness and chamber dimensions were measured. For each mouse, three to five values for each measurement were obtained and averaged for evaluation. Two research technologists trained in echocardiography and blinded to the experimental groups performed the studies. Left ventricular end-diastolic dimension (LVEDD), left ventricular end-systolic dimension (LVESD), interventricular septal wall thickness at end diastole (IVSD), and LVPW thickness at end diastole (LVPWTED) were measured from the M-mode tracing. LV fractional shortening (FS), the percent change in left ventricle cavity dimensions, was calculated using the following equation: fractional shortening (%) = [(LVEDD - LVESD)/LVEDD] × 100. Ejection fraction (EF) represents stroke volume as a percentage of end

diastolic LV volume and was calculated from the following equation: ejection fraction (%) = $[(LVEDD^2 - LVESD^2)/LVEDD^2] \times 100$. The heart rate was determined by counting the diastole and systole cycles during M-mode imaging within a defined time interval and multiplying by the correction factor to obtain heartbeats per min.

Histology

Cardiac histopathology was assessed in each treatment group based on the method of Billingham [3] as modified by Gabrielson *et al.* [4]. The hearts were fixed in 10% phosphate-buffered formalin, embedded in paraffin, sectioned at a thickness of 3 µm, and stained with toluidine blue. The frequency and severity of myocardial lesions induced by doxorubicin was assessed by light microscopic examination. The changes were graded on the basis of the number of cardiomyocytes showing necrosis, mineralization, and cytoplasmic vacuolization.

Immunohistology

Immunohistochemistry was performed on formalin-fixed paraffin-embedded tissue, using common lab techniques. Briefly, the slides were deparaffinized using xylenes and hydrated by a graded series of ethanol washes. Antigen retrieval was accomplished by heating the slides in citrate buffer (pH 6.0) at 90°C for 20 minutes. Endogenous peroxidase activity was quenched by 10 min incubation in 3% H_2O_2, and nonspecific binding was blocked by incubation in 10% fetal bovine serum solution (Invitrogen, Carlsbad, CA) before incubation with the primary antibody. Chromogenic detection was enabled using the PowerVision+ Poly-HRP IHC kit (Immunovision Technologies, Norwell, MA) following the manufacturer’s protocol. Slides were counterstained with Harris-hematoxylin solution. Primary antibodies utilized were: anti-p65 (dilution 1:200), anti-MIB-1 (Ki-67) (dilution 1:100). Quantification of signal was performed by evaluating 10 random high power fields (40X magnification) on each slide, and counting the total number of cells with positive labeling. In the case of Ki-67, only nuclear localization of chromogenic signal was

counted as positive. Four independent xenografts were evaluated for each treatment condition.

Fluorometric TUNEL assay was performed according to manufacturer's protocol (Promega).

References

1. Sahn DJ, DeMaria A, Kisslo J and Weyman A. Recommendations regarding quantitation in M-mode echocardiography: results of a survey of echocardiographic measurements. Circulation. 1978; 58(6):1072-1083.
2. Yang XP, Liu YH, Rhaleb NE, Kurihara N, Kim HE and Carretero OA. Echocardiographic assessment of cardiac function in conscious and anesthetized mice. The American journal of physiology. 1999; 277(5 Pt 2):H1967-1974.
3. Billingham M. (1991). Role of endomyocardial biopsy in diagnosis and treatment of heart disease. In: Silver M, ed. Cardiovascular Pathology. (New York: Churchill Livingston), pp. 1465–1486.
4. Gabrielson KL, Hogue BA, Bohr VA, Cardounel AJ, Nakajima W, Kofler J, Zweier JL, Rodriguez ER, Martin LJ, de Souza-Pinto NC and Bressler J. Mitochondrial toxin 3-nitropropionic acid induces cardiac and neurotoxicity differentially in mice. The American journal of pathology. 2001; 159(4):1507-1520.
